# PRINS, a primate-specific long non-coding RNA, plays a role in the keratinocyte stress response and psoriasis pathogenesis

**DOI:** 10.1007/s00424-016-1803-z

**Published:** 2016-03-03

**Authors:** Márta Széll, Judit Danis, Zsuzsanna Bata-Csörgő, Lajos Kemény

**Affiliations:** Department of Medical Genetics, Faculty of Medicine, University of Szeged, Szeged, Somogyi B. u. 4, 6720 Hungary; MTA-SZTE Dermatological Research Group, Szeged, Korányi fasor 6, 6720 Hungary; Department of Dermatology and Allergology, Faculty of Medicine, University of Szeged, Szeged, Korányi fasor 6, 6720 Hungary

**Keywords:** Psoriasis, lncRNA, Keratinocyte stress response, PRINS, Primate specific

## Abstract

In the last few years with the recent emergence of high-throughput technologies, thousands of long non-coding RNAs (lncRNAs) have been identified in the human genome. However, assigning functional annotation and determining cellular contexts for these RNAs are still in its infancy. As information gained about lncRNA structure, interacting partners, and roles in human diseases may be helpful in the characterization of novel lncRNAs, we review our knowledge on a selected group of lncRNAs that were identified serendipitously years ago by large-scale gene expression methods used to study human diseases. In particular, we focus on the Psoriasis-susceptibility-Related RNA Gene Induced by Stress (PRINS) lncRNA, first identified by our research group as a transcript highest expressed in psoriatic non-lesional epidermis. Results gathered for PRINS in the last 10 years indicate that it is conserved in primates and plays a role in keratinocyte stress response. Elevated levels of PRINS expression in psoriatic non-lesional keratinocytes alter the stress response of non-lesional epidermis and contribute to disease pathogenesis. Finally, we propose a categorization for the PRINS lncRNA based on a recently elaborated system for lncRNA classification.

## Introduction

One of the biggest surprises at the completion of the Human Genome Project [24] was the discovery of the low protein-coding capacity of the completed sequence: only approximately 2 % of our genome encodes proteins, corresponding to roughly 20,000 genes. Is it a matter of wastefulness that “Mother Nature” maintains at least 234 genes (more than 1 % of protein encoding genes) [41] to keep our genome in a good shape when only 2 % of it has any meaning? Now, it is clear that this repair machinery is not working leanly, as approximately 80 % of our DNA content is functional and most of it is transcribed into RNA [20, 22]. Understanding this only opened new questions: what is the function of this large amount of RNA? Will this understanding bring us closer to resolving questions about cell physiology? Moreover, will it bring new understanding of the still unknown mechanisms of genetically determined diseases that afflict us? Thanks to recently developed molecular biology tools, such as tiling array technologies [46] and RNA-Seq [13] as well as bioinformatics tools [8], we know that tens of thousands of functional RNA molecules are transcribed from our genome with varying length, genomic content, and functions. Concomitant with the growing number of characterized RNA molecules, the need for effective classification has increased. Although many attempts have been made to define subclasses of RNA function and various classification criteria have been proposed by multiple authors [62], RNA size has remained the primary aspect amenable for classification. From the start of the classification attempts, it was suspected that microRNAs (miRNAs, approximately 22 nucleotides in length) comprised a distinct class of molecules within non-coding RNAs. Now, it is clear that their function, as well as their size, distinguishes miRNA. To date (http://www.mirbase.org/cgi-bin/mirna_summary.pl?org=hsa), 1881 distinct human miRNAs have been identified, and since 2001, extensive research has uncovered the function of many miRNAs in physiologically normal and disease conditions. miRNAs are known to be involved in the regulation of all aspects of intracellular events and the mechanism by which they act has been clarified [23].

The function of another distinct group, the long non-coding RNAs (lncRNA, >200 nucleotides), is not as well known. Thousands of lncRNAs have been identified in the recent years [74], and it is generally accepted that this group of RNAs has much more diverse functions than miRNAs.

Already at the beginning of the molecular biology revolution, it was relatively easy to identify protein-coding genes within human sequences by identifying open reading frames (ORF). No such sequence analysis tools are available for identifying non-coding genes. Since the 1990s, when the importance of lncRNAs was uncovered, many attempts have been made to develop computational approaches for identifying lncRNAs within the human genome [25, 75]. Through the efforts of the GENCODE Project, a good estimate for the frequency, the structure, the evolution, and the expression of lncRNAs is available [17]. An analysis of lncRNA annotation by Darrien et al. (2012) concluded that the human genome includes approximately 14,000 lncRNA genes, a number that are much higher than those estimated for miRNA genes and relatively close to the number of protein-coding genes. Although much information has been gathered about the functions of proteins and miRNAs in health and in disease, we are still at the beginning of our journey in exploring the functions of lncRNAs.

## The great past and bright future of lncRNAs unexpectedly identified by large-scale gene expression experiments in human diseases

In order to understand the functions of thousands of lncRNAs, we must characterize them one by one, understand their molecular and cellular contexts, and discover their contribution to human diseases. Before the “big boom” of omics research, sporadic reports of the identification and functional characterization of lncRNAs were available. Interestingly, most of these early reports came from attempts to identify differentially expressed transcripts in human diseases using relatively old-fashioned methods of large-scale gene expression, such as complementary DNA (cDNA) λ phage library screening [16], cDNA subtractive hybridization [33], and differential display [61]. These studies identified the first lncRNAs, such as BC200, MALAT-1, PCGEM, and DD3. In subsequent systematic studies, much information has been gathered on structures, interaction partners, subcellular localizations, and functions of these lncRNAs. Moreover, data clearly indicated that these gene products might serve as novel diagnostic tools and therapeutic targets for treating human diseases. Clearly, lncRNA research is not just an “l’art pour l’art” activity of biomedical specialists but is expected to have clinical utility.

Two important lncRNAs early identified were BC1, a neuron-specific rodent lncRNA [16], and its human orthologue, BC200 [70]. The in-depth study of these two orthologues revealed interesting data on their functions, long before the upheaval brought by the advent of systematic large-scale bioinformatics and next-generation techniques. These lncRNAs are expressed in neurons, specifically in the post-synaptic area of dendrites. Both gene products are part of a cytoplasmic ribonucleoprotein complex and bind to the poly(A)-binding protein (PABP) [43, 48]. PABP plays an important role in translation regulation in eukaryotic cells, along with translation factors, such as eIF4F, eRF3, and Paip proteins [36, 57]. Furthermore, it has been also reported that BC1 and BC200 bind the fragile X mental retardation protein, the product of the FMRP disease-causing gene [79]. Dysfunction(s) of BC1/BC200-FMRP binding leads to abnormal translation in the post-synaptic area and contributes to the phenotype seen in fragile X mental retardation syndrome [80]. BC1 and BC200 were the first lncRNAs to be used in knock-out experiments: BCI knock-out mice exhibited behavioral changes and shorter life spans compared to controls [45]. BC200 was also shown to have a role in aging and Alzheimer’s disease: Mus et al. (2007) [50] have demonstrated that normal aging is associated with decreased BC200 expression in the brain, while BC200 expression was significantly upregulated in the brains of Alzheimer’s patients, where abnormal localization to the perykarial region was observed. Under normal conditions, BC200 is expressed exclusively in neurons; however, BC200 was found to be expressed in human neoplasms [10]. Recently, De Leener et al. [15] proposed BC200 as a potential biomarker for diagnosing early-stage breast cancer.

BC200 is not the only early-identified lncRNA that holds promise as a diagnostic marker in human cancer. The prostate-specific PCGEM1 and DD3 lncRNAs were identified by differential display as early as 2000 [61]. PCGEM1 was found to have ethnic-specific expression, being much higher in the prostate epithelial cells of African-American prostate cancer patients compared to Caucasian patients. Moreover, PCGEM was found to be upregulated in normal prostates of individuals with relatives who had been diagnosed with prostate cancer. The pathogenic role of PCGEM1 in prostate cancer was further indicated by its ectopic expression in LNCaP and NIH3T3 cells, which resulted in hyperproliferation [53]. Some years later, PCGEM1 was patented as a promising biomarker for prostate cancer [32]. In a recent paper, it was suggested that PCGEM may serve as a reliable biomarker for the assessment of drug efficacy during prostate cancer treatment [29]. While PCGEM is expressed both in healthy and in diseased prostates, the DD3 lncRNA, identified in the same differential display experiment [61], exhibits strictly specific expression for prostate cancer, providing the possibility of developing a highly specific diagnostic tool for the disease. Based on this observation, Tinzl et al. (2004) [71] developed a nucleic acid-based diagnostic tool that can be used to detect DD3 lncRNA from urine with high specificity and sensitivity. The product, Progensa, has been on the market since 2011 [21]. In a recent advance, Ding et al. [19] developed an oncolytic viral vector, Ad.DD3.E1A.E1B(Δ55)-(PTEN), under the control of the prostate-specific DD3 promoter and proved its apoptotic effect in prostate cancer cell lines.

MALAT-1 lncRNA was first identified in a cDNA subtractive hybridization experiment from metastasizing lung adenocarcinoma in 2000 [33]. MALAT-1 is possibly the most extensively studied of the early-identified lncRNAs, as it is expressed in a wide range of tissue types and its over-expression has been detected in many human malignancies. Extensive studies have also shed light on the cellular function of MALAT-1: Hutchinson et al. (2007) [31] reported that MALAT-1 co-localizes with SC35 splicing domains, suggesting that MALAT-1 may be a component of the splicing machinery. *Cis* and *trans* regulatory factors for the localization of the MALAT-1 lncRNA to nuclear speckles have been identified [47]. Functional studies performed on cell lines from different types of human malignancies revealed that MALAT-1 is indeed key for the maintenance of hyperproliferation and metastasizing potential [26, 34, 56, 69, 76, 77, 81, 83]. In addition to being a promising biomarker for the diagnosis of a wide range of human malignancies, MALAT-1 proved to be a putative target for siRNA-mediated therapy, as recently demonstrated by Ren et al. [54].

Taken together, the above examples of lncRNAs demonstrate well that several gene products incidentally identified by large-scale gene expression studies have been scientifically and medically interesting, and their study has not only lead to a better understanding of human pathologies but has uncovered potential diagnostic tools and therapeutic targets. Next, we discuss the example of an lncRNA identified by differential display in a study of psoriasis. We describe its role in keratinocyte physiology and psoriasis pathogenesis.

## Identification of PRINS, an lncRNA involved in psoriasis pathogenesis

Psoriasis, affecting approximately 2–4 % of the population, is a classic multifactorial skin disease. The interplay of multigenic susceptibility as well as environmental and lifestyle factors leads to the development of symptoms, characterized by epidermal hyperproliferation and inflammation [18]. Intensive research of the last few decades revealed that abnormally functioning keratinocytes and skin-infiltrating professional immune cells are responsible for the disease phenotype [4–6]. As yet, it is still unknown whether abnormal keratinocyte functions of normal-appearing epidermis or aberrant lymphocyte activation is the primary trigger for development of the disease. Accumulating evidence suggests that altered skin tissue homeostasis, especially keratinocyte-specific alterations of the normal-appearing skin of psoriatic patients, is key in the initiation of the disease phenotype. The “immune era” of psoriasis research unquestionably brought breakthroughs for new, targeted therapies of the disease [28]. Nonetheless, to identify novel targets for intervention and possibly for prevention, we must understand the role of aberrant keratinocyte function in the course of the disease.

To this end, the aim of our workgroup is to identify and characterize abnormal molecular patterns in non-lesional psoriatic keratinocytes contributing to the initiation of the disease phenotype and factors that make these keratinocytes prone to respond with hyperproliferation to cytokines produced by skin-infiltrating lymphocytes. We previously performed a differential display experiment to compare gene expression in non-lesional psoriatic epidermis and control healthy epidermis. In 2000, several differentially expressed protein-coding transcripts in the psoriatic non-lesional epidermis were identified, and of these, we focused on the expression of EDA+ fibronectin. We were first to demonstrate that, upon cytokine induction, keratinocytes of the non-lesional epidermis are able to produce this form of cellular fibronectin and, thus, maintain an autocrine loop resulting in keratinocytes hyperproliferation [66]. This finding confirmed our a priori hypothesis that not only professional immune cells, but also keratinocyte-derived factors contribute to disease susceptibility.

In addition to protein-coding transcripts differentially expressed in psoriatic non-lesional epidermis, we also identified a transcript that was unlikely to encode a protein but, nevertheless, exhibited 100 % sequence identity to the 3′ end of a cDNA (GenBank accession number AK022045) previously identified in a cDNA library derived from a 10-week-old human embryo. In parallel with sequence similarity searches, in vitro experiments were performed to determine the expression pattern of the identified transcript during proliferation and differentiation of keratinocytes. The highest expression of the non-coding RNA was found in serum-starved, contact-inhibited keratinocytes as well as in these cells immediately after serum re-addition; however, when the cells began to proliferate, the expression of the AK022045 transcript dropped dramatically. With this compelling result, we decided to turn our focus to the in-depth characterization of this transcript, and since then, we have been engaged in parallel but manifold interconnected characterization of its role in psoriasis and in keratinocyte stress response.

Extensive sequence similarity searches and the determination of the 5′ end of the transcript allowed us to draw a putative structure for the newly identified gene. The full-length transcript is 3.6 kb, and a putative TFIIB transcription factor binding site was identified in the genomic sequence proximal to the putative transcription start site. The transcript contains two exons, both harboring *Alu* elements, and shows a high degree of similarity to a heat shock element in a small non-coding RNA, *G8*. Based on these findings, we supposed that this transcript is an lncRNA and named it Psoriasis-susceptibility-Related RNA Gene Induced by Stress (PRINS). The full-length sequence is available to the scientific community (http://www.ncbi.nlm.nih.gov/gene/?term=PRINS[sym]) [58].

By using quantitative real-time PCR (Q-RT-PCR) [58] and in situ hybridization (ISH) methods [65], PRINS expression was determined in human tissue types. These two experimental approaches revealed that PRINS expression varied in different human tissue types: the highest level of expression was observed in the veins, high levels were found in tissues derived from female and male gonads and lung, moderate expression was detected in tissue types derived from the gastrointestinal tract, and no apparent expression was present in the brain. Both ISH and Q-RT-PCR revealed a relatively high level of basal PRINS expression in healthy epidermis.

The contribution of PRINS to the pathogenesis of psoriasis susceptibility was further indicated in experiments of organotypic skin cultures. Organotypic skin cultures from healthy volunteers and from the non-lesional skin of psoriatic patients were co-incubated with a T cell lymphokine mix previously shown to induce the proliferation of non-lesional psoriatic epidermal keratinocytes but not keratinocytes derived from normal healthy epidermis [5, 6]. PRINS expression differed in the two systems: while the treatment decreased PRINS expression in the non-lesional psoriatic epidermis, it was unchanged in the normal healthy epidermis. This result suggested that PRINS may contribute to psoriasis susceptibility as a modifier gene and may be part of the inherently altered molecular network of non-lesional epidermal keratinocytes contributing to disease pathogenesis [58].

To identify PRINS interacting partners and targets, in vitro binding assays [65] and cDNA microarray experiments [64] were performed. In the latter, PRINS expression was silenced in keratinocytes and the genes with altered expressions were studied in detail. G1P3, one of the identified genes to be under the control of PRINS, had been previously shown to play a pathogenic role in human malignancies with anti-apoptotic effects, and it is regulated by interferon-α [11, 67]. These two features of G1P3 are also important in psoriasis pathogenesis [40, 52]. We found that the mRNA expression of PRINS-regulated G1P3 was upregulated 400-fold in lesional and 9-fold in non-lesional psoriatic epidermis, compared to healthy epidermis. In vitro experiments revealed that the down-regulation of G1P3 inhibited the spontaneous apoptosis of keratinocytes, indicating that its high expression might contribute to the altered apoptotic features of psoriatic keratinocytes and, thus, to disease pathogenesis. Taken together, these results indicate that the deregulation of the PRINS lncRNA contributes to psoriasis pathogenesis at least partially by altering the expression of G1P3 and leading to decreased sensitivity of keratinocytes toward spontaneous apoptosis [64]. In another set of experiments, an in vitro binding assay identified the nucleophosmin (NPM) protein as a direct interacting partner of the PRINS lncRNA. To determine whether this interaction had any relevance to psoriasis pathogenesis, the expression of NPM was studied in both healthy and psoriatic non-lesional epidermises. No apparent difference was found in the level or pattern of expression. Our finding was in agreement with a previous study examining nuclear staining for NPM in epithelial cells [7]. Additional evidence that NPM and the PRINS lncRNA might be direct interacting partners came from staining patterns in cultured keratinocytes: ISH staining of PRINS and immunohistochemical (IH) staining of NPM showed for both a predominant nuclear localization. In psoriatic lesional epidermis, however, the staining pattern of NPM was dramatically changed in the different layers of the epidermis: the highest level of NPM expression was found in basal and immediate suprabasal keratinocytes. Moreover, keratinocytes showing the highest level of NPM expression exhibited a marked cytoplasmic immunopositivity, revealing that, in keratinocytes of lesional psoriatic epidermis, both the level and the intracellular pattern of NPM expression were changed [65].

Thus, altered expression of proteins that are either interacting partners of PRINS or are under the control of PRINS indicates that, indeed, this lncRNA plays an important role in psoriasis and that, by altering normal keratinocyte function(s), it contributes to disease pathogenesis.

## The role of PRINS in keratinocyte stress response

The first outstanding finding about the possible cellular functions of PRINS was the contrast between the high expression found in serum-starved, contact-inhibited keratinocytes and the very low levels of expression in highly proliferating keratinocytes [58], suggesting that PRINS may have a key role in the keratinocyte stress response. To test this, the survival of keratinocytes was studied during down-regulation of PRINS. No effects of PRINS down-regulation were found on the survival of keratinocytes under favorable culturing conditions; however, when serum was withdrawn from the PRINS down-regulated keratinocytes, the survival rate decreased significantly. This result confirmed that elevated PRINS expression in stressed keratinocytes is not an epiphenomenon but that, indeed, this lncRNA contributes to the cellular stress response. Further in vitro experiments revealed that other stress factors, including microbial components (viral and bacterial), translation inhibition with cyclohexamide, and UV-B irradiation, are able to induce high-level PRINS expression in keratinocytes. The UV-B results were especially intriguing, since the PRINS interaction partner NPM has a well-documented role in cellular UV-B response: the predominantly nucleolar localization of NPM is changed upon UV-B irradiation in fibroblasts, and in cancer cells [44, 78], translocation to the nucleoplasm and, to some extent, to the cytoplasm occurs. In vitro experiments were performed to determine whether the same phenomenon occurs in epidermal keratinocytes and whether PRINS has any role in it. PRINS expression was down-regulated in UV-B-irradiated keratinocytes, and intracellular re-distribution of NPM occurred subsequently. Thus, NPM was localized to the nucleus in keratinocytes in which PRINS was down-regulated, indicating that PRINS physically interacted with NPM as well as functionally regulated NPM in this cellular stress response [65]. Taken together, our in vitro results suggested that PRINS lncRNA contributes to both stress responses and apoptosis signaling in keratinocytes, and relevantly, its role in psoriasis pathogenesis involves altering these pathways.

Nuclear factor kappa B (NF-κB) signaling is known to be altered in psoriatic keratinocytes [73], and this may be a link between T cell-mediated and keratinocyte-mediated processes in disease pathogenesis. To determine whether PRINS has a role in NF-κB signaling, PRINS expression was altered in keratinocytes and the activity of the NF-κB pathway was examined after lipopolysaccharide (LPS) induction and priming with psoriasis-related cytokines and detected using a luciferase-based approach. LPS induction of the NF-κB pathway is well known [49], and it has been shown that LPS can upregulate the expression of PRINS in keratinocytes. Neither the down-regulation [3] nor the robust upregulation [14] of PRINS had an effect on NF-κB activity, indicating that PRINS is not involved in this signaling pathway in keratinocytes, although other influences were not excluded.

We are currently working on identifying the signaling pathways in keratinocytes by which PRINS affects cell functions and contributes to disease pathogenesis in psoriasis.

## Classification of PRINS, a novel non-coding RNA conserved in primates

In the 1990s and early 2000s—when lncRNAs were identified accidently rather than by systematic search—the need to classify non-coding RNAs was not particularly compelling. Although the reports of novel lncRNAs caught the interest of the scientific community at scientific meetings, the general response was to consider them too eccentric to be taken seriously. However, this attitude has dramatically changed in the last few years as a result of two developments: on one hand, a large body information about lncRNAs has accumulated from large-scale gene expression studies in the last 15–20 years [35], and on the other hand, thousands of novel human lncRNA genes have been identified using high-throughput methods in the last few years [27]. Systematic annotation of these newly identified lncRNA transcripts was necessary to aid researchers in their search of the understanding the functions of lncRNAs.

In the last few years, several attempts have been made to address this need, resulting in a rather complicated system for categorizing aspects [62] as well as an “easier-to-follow” system for categorizing lncRNAs [38]. The varying—and we believe—still evolving taxonomy of lncRNA reflects the novelty of the field.

To provide a guide to the lncRNA classification suggested by Kapusta et al. [38], we use this system to apply an initial classification for the PRINS lncRNA.

According to the Kapusta classification, the first aspect to consider when categorizing an lncRNA is its genomic context. The first report of PRINS lncRNA [58] provided its location to be on the short arm of human chromosome 10 (map position 10p12.31) and indicated that it is composed of two exons and an intron of approximately 7 kb in length. The entire PRINS lncRNA resides in an intron of the recently annotated KIAA1217 gene, also known as SKT, which is involved in early stages of embryogenesis [63]. Proximal to KIAA1217 is the OUT deubiquitinase 1 gene, whereas distal to KIAA1217 is the Rho GTPase-activating protein 21. Interestingly, the miR603 miRNA is located in a KIAA1217 intron, 3′ of the PRINS coding region.

The second aspect to consider when categorizing an lncRNA is its chromatin context. A transcription start site was identified 6 kb proximal to the putative 5′ end of the PRINS gene using the ENCODE database. This region is marked by a high density of binding sites for several transcription factors, including GATA2, Fos, HDAC2 and STAT3, and histone modification sites associated with active transcription, such as mono- and tri-methylation of lysine 4 of histone H3 (H3K4me1/3) and acetylation of lysine 9 and 27 (H3K9Ac, H3K27Ac), suggesting that a strongly regulated active promoter might be associated with the lncRNA. The region adjacent to the 3′ end of the PRINS lncRNA gene also contains histone modification sights, which, due to the close 3′ proximity to the PRINS lncRNA gene, might be an enhancer element. Of all cells examined to date, the highest signals were found in normal human keratinocytes, indicating that the PRINS lncRNA is indeed expressed in keratinocytes and its expression is regulated by epigenetic factors.

The third and possibly most informative aspect to consider is the subcellular localization of the lncRNA. From our experimental results, we found that the PRINS lncRNA is mainly localized in the nucleolus of normal human cultured keratinocytes, although moderate perinuclear and cytoplasmic expression was also detected. This is in good agreement with reports that non-coding RNAs localize mainly to the nucleus [37], which suggests a role in the temporal–spatial regulation of nuclear organization and/or regulation of gene expression. The intracellular localization of the NPM protein, which was identified to be physically and functionally interacting with PRINS, is indicative of a nucleolar/nuclear role for PRINS. As early as 1984 [60], NPM was reported to be localized in the nucleolus and shown to shuttle between the nucleolus and the cytoplasm [9]. As both NPM [2] and PRINS [68] demonstrate strict regulation during cell growth and during cellular stress response and, additionally, have functionally overlapping features during these processes [65], we postulate that, indeed, these two molecules interact in these processes.

**Fig. 1:**
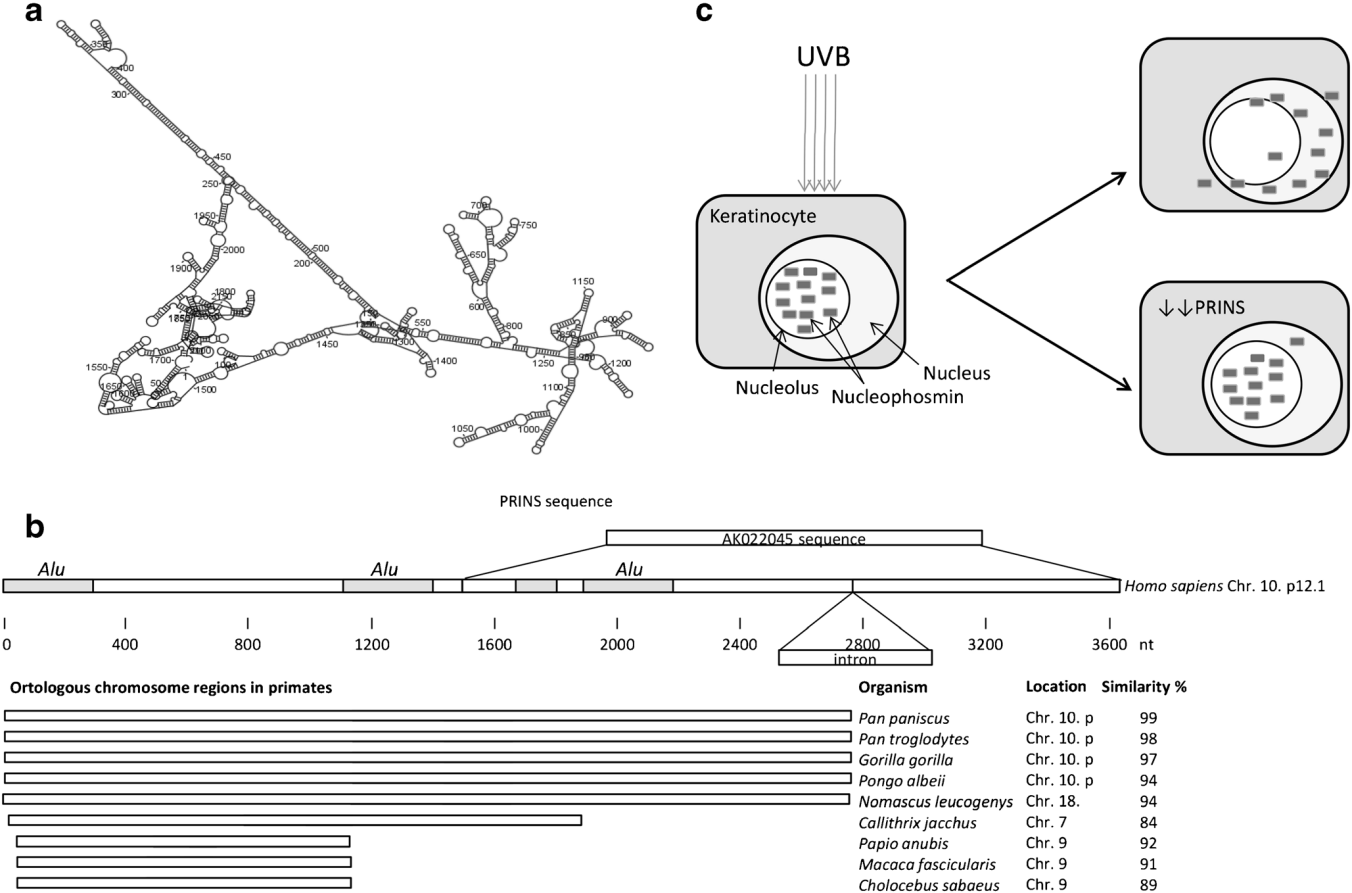
Major characteristics of the PRINS lncRNA. **a** Putative secondary structure of the PRINS lncRNA. **b** Similarity search identified three *Alu* elements within the PRINS lncRNA sequence. The PRINS gene is localized on the p12.1 arm of human Chr. 10, which is highly conserved in human and four other primate species. Although partial sequence similarity was found, it is largely due to the conservation of *Alu* elements. In other primate species, the PRINS sequence was distributed on other chromosomes. **c** UVB irradiation induces the shuttling of nucleophosmin (NPM) from the nucleolus to the nucleoplasm. Silencing of the PRINS lncRNA, which physically interacts with NPM, results in the retention in the nucleolus

Since sequence conservation of lncRNAs is rare, it is assumed that their biological activities are dependent on structure. The putative secondary structure of the PRINS lncRNA was determined from the primary sequence by computational prediction (Fig. 1a). In addition to strong structural features, the PRINS sequence might also determine its cellular localization, as it includes the AGCCC pentamer with the sequence restrictions at positions −8 (T or A) and −3 (G or C) of a motif which was reported to be crucial for nuclear localization of lncRNAs [82]. Our ISH results are in good agreement with this sequence-based analysis, as the highest level of PRINS expression was detected in the nucleolus of keratinocytes, with moderate staining in the nucleoplasm and the cytoplasm.

Intriguingly, the PRINS lncRNA sequence contains three *Alu* elements. It is well established that transposable elements (TEs) have been very important in the evolution of lncRNAs [39]. According to some estimates, approximately two thirds of functioning human lncRNAs contain at least one TE-derived element, which are seldom found in protein-coding genes. These elements very often make up a relatively large portion of the lncRNA genes [42]. This is also true for the PRINS lncRNA, which contains three *Alu* elements comprising approximately one third of its sequence (Fig. 1b). The possible contribution of TE elements to lncRNA evolution and function is extensively reviewed by Kapusta et al. [38].

It has been estimated that only 21 % of known lncRNAs occurring in primates have orthologues in other orders and only 3 % of the primate lncRNAs have an orthologue in tetrapods [51]. The results obtained with the PRINS lncRNA sequence agree well with these estimates, in that it could be found only in the genomes of primates with variations in the extent of similarity (Fig. 1b). PRINS and the orthologues with the highest similarity among primate species reside on the short arm of chromosome 10. Taken together, these data suggest that the PRINS lncRNA gene is most probably a primate-specific sequence and transposition was the major mechanism of its origin.

As many lncRNAs serve as sources for miRNAs [55], it was interesting to examine the PRINS lncRNA for miRNA pre-sequences. However, no pre-miRNA sequences were identified using the ever-growing mirbase database (http://www.mirbase.org/). It is interesting to note, however, that the intron in which the PRINS lncRNA is located harbors a miRNA. Analysis of miRNA harboring lncRNAs has shown it to be an evolutionary conserved group [12]. Thus, as PRINS is a primate-specific lncRNA, it is not surprising that it does not harbor any miRNA sequences.

The final criterion for classifying lncRNAs is the most challenging: function. However, this aspect is the most likely to advance the understanding of the role of lncRNAs in health and disease. Because the highest PRINS expression was observed in the nucleolus of normal human keratinocytes and the sequence contains a nuclear-specific motif, it is reasonable to assume its functions are in the nucleolus or nuclei. Our in vitro binding assays identified NPM as the most prominent interaction partner of PRINS, and the highest NPM expression was found in the nucleolus [60]. These results together with the results from the PRINS IHS and binding assay support the hypothesis that PRINS is a physical partner of NPM in the nucleolus and the complex formed by their binding contributes to the cellular stress response (Fig. 1c). Whether PRINS is interacting with proteins other than NPM and how it functions in concert with other keratinocyte-expressed lncRNAs [30] and/or miRNAs [59] are still challenging questions to answer. Moreover, the data cited above have been obtained from experiments performed in normal human keratinocytes and keratinocyte cell lines, leaving open the question whether PRINS is expressed with the same intracellular pattern and functions the same way in other cell types.

Together, the results from our experiments indicate that the evolutionarily young, primate-specific PRINS is one of the lncRNAs that is differentially expressed in psoriasis [72], that it plays a role in keratinocyte stress response, and that its elevated expression in psoriatic non-lesional epidermis contributes to the altered stress response of psoriatic keratinocytes and, thus, to disease pathogenesis. Psoriasis is a human-specific multifactorial skin disease, which has not yet been identified in other primates. It remains to be determined whether any association exists between the primate/human lncRNAs and the special susceptibility to certain multifactorial diseases that exist only in humans.

## Conclusions

There is no doubt that one of the greatest (and previously unforeseen) achievements of genome programs was the discovery of novel layers of cell regulation represented by lncRNAs. The importance of these transcripts is underscored by the fact that they are almost equal in number with protein-coding genes in the human genome [17]. We are still far from being able to comprehensively place these novel regulatory molecules into regulatory networks, similarly to what are currently known of protein–protein and protein–DNA interactions based on experimental data. A tremendous amount of experimental work, spanning decades, was necessary to gain sufficient information about individual proteins and DNA elements to describe these regulatory networks. Although highly developed in silico methods may speed this process for regulatory RNAs—including lncRNAs—it is reasonable to assume that, unless newly identified lncRNAs are experimentally characterized, it will not be possible to identify their cellular contexts. Classification of lncRNAs is crucial for their annotation, and information already available for the well-known, serendipitously identified lncRNAs is likely to have great importance in this work. According to some estimates, there are approximately 130 human lncRNAs extensively characterized to date [1] and they may serve well in the development of classification and annotation of thousands of identified but not yet characterized lncRNAs. In addition to understanding the cellular functions of these molecules, their contribution to human diseases will have to be elucidated, promising insights into the missing heritable and yet unrevealed mechanisms of human diseases.
